# Some New and Interesting Points in Ships' Hygiene

**Published:** 1912-06

**Authors:** 


**Some New and Interesting Points in Ships' Hygiene.** By W. Melville-Davison, M.B., B.S. Pp. 87. Bristol: John Wright & Sons Ltd. 1911.

This is a practical little book which gives the author's experience in regard to many points of hygiene of interest and importance on board ship. Mosquitoes, rats and other vermin, filters, disinfection, all receive consideration. Those who go down to the sea in ships should consult the volume.

